# Factors associated with quality of life in Chinese people with psoriasis: a cross-sectional study

**DOI:** 10.1186/s12889-023-16758-6

**Published:** 2023-09-25

**Authors:** Xiu-jie Zhang, Jing-rong Lin, Min-xing Ou, Hong-wei Yan, Sheng-nan Liu, Lu Dai, Fu-qing Gong

**Affiliations:** 1 School of Public Health, China Medical University, Shenyang, China; 2 Department of Dermatology, First Affiliated Hospital of Dalian Medical University, Dalian, China; 3 Department of Dermatology, First Affiliated Hospital of China Medical University, Shenyang, China; 4 Department of Dermatology, Dalian Dermatology Hospital, Dalian, China; 5 Department of Dermatology, Shenyang Dermatology Hospital, Shenyang, China

**Keywords:** Psoriasis, Quality of life, Anxiety/depression, Sleep disorders

## Abstract

**Background:**

The ultimate goal of medical care is to eradicate disease and restore normality to a person’s life. Quality of life (QOL) is a concern as dermatologists and researchers strive to find better drug treatments. However, there have been few reports on the factors associated with QOL among Chinese people with psoriasis.

**Methods:**

A total of 185 people with psoriasis were surveyed to assess their sociodemographic status, disease-related information, psychosocial status, and QOL. The questionnaires included a sociodemographic questionnaire, the Athens Insomnia Scale, the Hospital Anxiety and Depression Scale, the Perceived Social Support Scale, the Psychosocial Adaptation Questionnaire of Chronic Skin Disease and the Dermatology Life Quality Index. Multiple stepwise regression and path analysis were used to study the factors associated with QOL among Chinese people with psoriasis and to analyse the relationship between them.

**Results:**

The results showed that the presence of anxiety/depression, lesion area, sleep disorders, psychosocial adaptation, and sex could jointly predict 62.1% of the variance in QOL among Chinese people with psoriasis. According to previous theories and the literature, a path model was established for five variables. Four internal variables could be effectively explained. The values of the explanatory variables were 62.1% (F(1056) = 61.020, *p* = 0.000) for QOL, 71.8% (F(2433) = 117.370, *p* = 0.000) for anxiety/depression, 44.0% (F(660) = 36.935, *p* = 0.000) for sleep disorders, and 66.9% (F(6886) = 93.556, *p* = 0.000) for psychosocial adaptation. The path analysis confirmed that 9 paths were consistent with the predicted path, and 3 paths were not confirmed.

**Conclusion:**

To improve QOL among Chinese people with psoriasis, attention should be given to the presence of anxiety/depression, lesion area, sleep disorders, psychosocial adaptation and sex differences. Therefore, health care programs for psoriasis should include physical, psychological and social aspects.

## Introduction

Psoriasis is a relapsing, chronic, immune-mediated common systemic inflammatory skin condition that affects more than 125 million people worldwide [1, 2]. Nearly 30% of patients with psoriasis develop significant comorbidities, including arthritis, cardiovascular disease, inflammatory bowel disease, chronic kidney disease, malignant tumours, infection, mental disorders, and metabolic syndrome [3, 4]. Psoriasis patients not only suffer from skin damage but also often suffer from physical and mental disorders due to skin integrity damage or low self-esteem, embarrassment, anxiety, and depression, which affects their quality of life (QOL) [5]. Most patients with psoriasis have some decline in their QOL attributable to the disease, and many experience a substantial, negative effect on their psychosocial wellbeing [6]. The QOL of people with psoriasis in China is generally low, which manifests in the following ways [7]. The mental burden is serious: 89% of the people with psoriasis in China report mental pressure, 78% experience discrimination, 34% have suicidal thoughts due to illness, 5% commit suicide, with only 11% seeking psychological treatment, and 51.5% choose to close themselves off to society, their friends, and even their family, to reduce the psychological pressure, which has become a substantial hidden danger that affects their health and social security [7]. The serious economic burden and treatment expenditure of most people with psoriasis accounts for 20% of their annual income, and the annual hospitalization rate is 13% (the hospitalization rate of moderate and severe patients is 26%) [7]. A total of 37% of people with psoriasis are unemployed due to illness, 73% of people with psoriasis are unable to work due to illness, and the unemployment rate of patients with moderate and severe psoriasis is as high as 48%; therefore, the social and economic burden caused by psoriasis should not be underestimated [7]. Psoriasis has undoubtedly become a serious public health problem [8].

Since the 21st century, medical science has been entering a new epoch of biology, and the medical model has shifted from a single biology model to a biopsychosocial model [9]. Some scholars have conducted qualitative and quantitative studies on the factors affecting the QOL of people with psoriasis, which has also been reviewed by researchers [10–16]. The influencing factors include demographic (age, sex, marital status, education level, alcohol consumption, smoking status, Psoriasis Area and Severity Index (PASI) score, clinical symptoms, visual position, lesion area, and course of disease) [11, 14, 17–21], psychological (anxiety/depression, sleep disorders) and social aspects [14, 15, 19, 22, 23]. Preliminary studies have found that these influencing factors show interactions in people with psoriasis [24]. However, the effects and mechanisms of the factors affecting QOL in people with psoriasis are rarely discussed.

On the one hand, single-factor analysis and multiple stepwise regression were used to determine the main factors impacting the QOL of psoriasis patients in China. On the other hand, based on previous theory and experience, a predicted path was established for path analysis to determine the relationships between the main factors.

## Methods

### Study design and setting

This was a cross-sectional study of 185 people with psoriasis conducted from May to October 2019 in China.

### Study population and sampling

A convenience sampling method was used to select outpatients and inpatients from 3 hospitals. The inclusion criteria were an age of at least 16 years and a time since the confirmed diagnosis of psoriasis of more than 1 month. The exclusion criteria were severe cognitive impairment, cardiovascular or other conditions that significantly affect QOL, and dropping out of the study during the study period. People with psoriasis were recruited by nurses in our research group and were invited to participate when they met for their appointment and complete a battery of paper-based questionnaires for data collection. Ethics approval was obtained by the Ethics Committee (Code YJ-KY-SB-2018–86). Following the Declaration of Helsinki, the purpose, content and confidentiality of the study were fully explained to the participants, who had the free choice to voluntarily agree or refuse to participate in the study. All participants gave their written informed consent. The sample size calculated is 5–10 times the number of variables [25]. The number of variables included in this study is 24, considering the 10% loss of follow-up rate, so the sample size is 132–264.

### Study instruments

The survey instruments, which were completed on paper, were as follows:

#### Sociodemographic questionnaire

The sociodemographic characteristics included demographic (age, sex, marital status, education level, alcohol consumption and smoking status) and disease-related variables (PASI score, clinical symptoms, visual position, lesion area, and course of disease).

#### Athens insomnia scale (AIS) [26]

The AIS was used to self-measure sleep quality, consisting of 8 items that are scored by using a 7-point Likert scale. A four-level scoring method of 0–3 was adopted, with a total score of less than 4 classified as no sleep disorder, a score between 4–6 as suspicious insomnia, and a score above 6 as insomnia. The Cronbach's α coefficient of this scale in this study population was 0.914.

#### Hospital anxiety and depression scale (HADS) [27]

In 1983, Zigmond AS and Snaith RP developed it to assess the anxiety and depression of hospital patients. The Chinese version was translated by WF Ye and JM Xu in 1993. It consists of 14 items, including 7 items for anxiety and depression, which can be scored separately. The subscales of anxiety and depression were divided into 0–7 (asymptomatic), 8–10 (suspicious symptoms) and 11–21 (symptoms). Its Cronbach's α coefficient in the study population was 0.908.

#### Perceived social support scale (PSSS) [28]

The PSSS was used to self-measure perceived social support and contains 12 items that are scored by using a 7-point Likert scale. The lowest scor is 12 points and the highest 84 points. The higher a patient’s score, the better their perceived social support. The Cronbach's α coefficient of this scale in the study population was 0.917.

#### Psychosocial adaptation questionnaire among patients with chronic skin disease (PSAQ-CSD) [29]

The PSAQ-CSD was used to assess the psychological adaptation of people with psoriasis and includes 18 items that are scored by using a 5-point Likert scale. The higher a patient’s score is, the better their psychosocial adaptation. The Cronbach's α coefficient of this scale in the study population was 0.934.

#### Dermatology life quality index (DLQI) [30]

The DLQI has been widely used to measure the QOL of people with CSD. It consists of 10 items that reflect health in terms of symptoms and feelings of CSD, daily activities, work and school, relationships, leisure and therapy. It is scored from 0 to 3 (a little, a lot, or very much). The Cronbach's α coefficient was 0.901. The DLQI score was classified as 0–1 (no affected), 2–5 (small affected), 6–10 (moderate affected), 11–20 (very large effect) and 21–30 (extremely large effect) [31].

### Statistical analysis

All statistical analyses were performed with the Software Package for Social Sciences for Windows v25.0 (IBM, Armonk, New York). Categorical data are presented as frequencies and percentages. Continuous data are presented as the mean ± standard deviation or median and interquartile range, as appropriate. To compare categorical data between groups, χ2 tests were performed. Continuous data were compared using the Mann‒Whitney U test or Kruskal‒Wallis H test, as appropriate. Statistically significant factors in univariate analysis (*p* < 0.05) were included in multivariable models. Stepwise multiple linear regression (SMLR) and path analysis (PA) were used to identify the major factors associated with quality of life. A two-tailed *p* value < 0.05 was considered statistically significant.

## Results

A total of 226 people with psoriasis who met the inclusion and exclusion criteria were recruited from wards and outpatient departments from May to October 2019, and 215 patients agreed to participate (response rate 95.13%). In the process of data entry, 30 substandard questionnaires were excluded (13 with incomplete basic information, 17 with missing items or repeated answers), and 185 effective questionnaires (effective rate: 86.05%) were ultimately obtained. The sociodemographic data of the participants are shown in Table 1. The DLQI score was 11.74 ± 6.74, 0–1 had no affected on 6 (3.24%), 2–5 had small affected on 32 (17.30%), 6–10 had moderate affected on 56 (30.27%), 11–20 had very large effect on 69 (37.30%) and 21–30 had extremely large effect on 22 (11.89%).

**Table 1:** Univariate analysis of QOL in people with psoriasis (*n* = 185)

Item	n (%)	Rank mean	Z/χ^2^	*p*	Item	n (%)	Rank mean	Z/χ^2^	*p*
**Nation**			-1.189	0.235	**BMI**			0.781	0.677
Han nationality	175	91.88			emaciation < 18.5	3	88.50		
Other	10	112.55			normal18.5–24.9	99	96.24		
**Sex**			-2.674	0.007	overweight ≥ 25.0	83	89.30		
Male	125	100.28			**Smoking status**			-2.076	0.038
Female	60	77.8			yes	58	105.08		
**Age**			0.305	0.858	no	127	87.48		
< 40	73	90.79			**Disease course**			11.232	0.004
40–59	65	93.08			< 12	17	52.15		
≥ 60	47	96.32			12–60	20	90.93		
**Marital status**			1.459	0.692	> 60	148	97.97		
Married	141	94.45			**Have you ever used folk remedies?**			-1.925	0.054
Unmarried	34	84.31			yes	95	100.37		
Divorce	6	107.75			no	90	85.22		
Widowed	4	93.75			**Visual position**			-3.370	0.001
**Alcohol consumption**			-0.249	0.803	yes	139	100.02		
Yes	54	91.47			no	45	69.28		
No	131	93.63			**Sensitive position**			-1.533	0.125
**Exercise**			5.509	0.064	yes	168	94.92		
Regular exercise	33	73.32			no	17	74.06		
Irregular exercise	118	97.88			**Lesion area**			22.849	0.000
Don’t exercise	34	95.16			< 20%	73	70.97		
**Education level**			5.461	0.141	20%-70%	60	100.11		
Junior high school and below	49	97.97			> 70%	52	115.73		
High school/technical secondary school	59	99.43			**Pain**			-3.954	0.000
Junior college	27	97.81			yes	124	82.10		
Bachelor degree or above	50	77.94			no	61	115.16		
**Occupation**			5.769	0.217	**Pruritus**			-2.304	0.021
Staff	56	83.84			yes	175	95.17		
Other	44	96.09			no	10	55.10		
Retire	44	87.99			**Anxiety/depression**			91.033	0.000
Worker	31	104.06			low grouping	51	44.28		
Farmer	10	118.45			middle grouping	82	90.54		
**Monthly household income (USD)**			3.640	0.303	high grouping	52	144.66		
< 695.62	76	86.84			**Social support**			29.804	0.000
695.62–1391.11	67	93.68			low grouping	51	117.54		
1391.12–2086.73	14	115.54			middle grouping	83	97.67		
More than 2086.74	28	96.82			high grouping	51	60.85		
**Pay**			3.090	0.378	**Sleep disorders**			67.024	0.000
Fully able to pay	41	99.93			low grouping	66	54.42		
Pay	59	90.48			middle grouping	68	98.72		
Grudgingly pay	63	75.46			high grouping	51	135.39		
Difficulty in paying	22	88.98			**Psychosocial adaptation**			75.908	0.000
**PASI score**			-8.834	0.000	low grouping	51	140.04		
Low grouping	52	26.50			middle grouping	84	91.60		
Middle grouping	80	92.50			high grouping	50	47.38		
High grouping	53	159.00							

The total scores of PASI, anxiety/depression, social support, insomnia and psychosocial adaptation were ranked from highest to lowest, and the scores at 27% above and below were found as the demarcations and divided into high, medium and low groups

### Univariate analysis of QOL in people with psoriasis

The rank sum test was used to analyse sociodemographic, disease, psychological and social factors, and it was found that sex, smoking status, disease course, visual position, lesion area, PASI score, pruritus, pain, anxiety/depression, sleep disorders, psychosocial adaptation and social support had statistically significant effects on QOL (*P* < 0.05), as shown in Table 1.

### Multiple stepwise regression analysis of QOL in people with psoriasis

The QOL of people with psoriasis was taken as the dependent variable, and the 12 variables with statistical significance in univariate analysis, including sex, smoking status, disease course, visual position, lesion area, PASI score, pruritus, pain, anxiety/depression, sleep disorders, psychosocial adaptation and social support, were taken as independent variables. There were 5 significant variables entered into the regression equation, the multivariate correlation coefficient was 0.795, and the joint explanatory variance was 0.621. These variables jointly predicted 62.1% of the variance in QOL, and anxiety/depression had the best predictive power (56.5%), followed by lesion area (2.9%), sleep status (2.1%), psychosocial adjustment (0.8%) and sex (0.8%). The standardized regression equation was as follows: QOL = 0.466 × anxiety/depression + 0.149 × lesion area + 0.168 × sleep disorders -0.163 × psychosocial adaptation -0.093 × sex. The collinearity diagnosis of the regression model showed that the VIF values of the 5 variables ranged from 1.045–3.620, λ was greater than 0.000, and the CI of the whole equation combination was 20.091, which confirmed that the equation combination was stable and had no collinearity problem (Table 2).

**Table 2:** Multiple stepwise regression analysis of QOL in people with psoriasis (*n* = 185)

Item	R	R Square	Standardized regression coefficient	F-value	*p*-value	VIF-value	λ-value
Anxiety/depression	0.752	0.565	0.466	237.765	0.000	3.620	0.757
Area of lesion	0.770	0.589	0.149	132.874	0.000	1.076	0.428
Sleep disorders	0.784	0.609	0.168	96.334	0.000	1.825	0.257
Psychosocial adaptation	0.789	0.615	-0.163	74.388	0.000	3.082	0.107
Gender	0.795	0.621	-0.093	61.328	0.000	1.045	0.011

### Pathway analysis of QOL in people with psoriasis

**Fig. 1:**
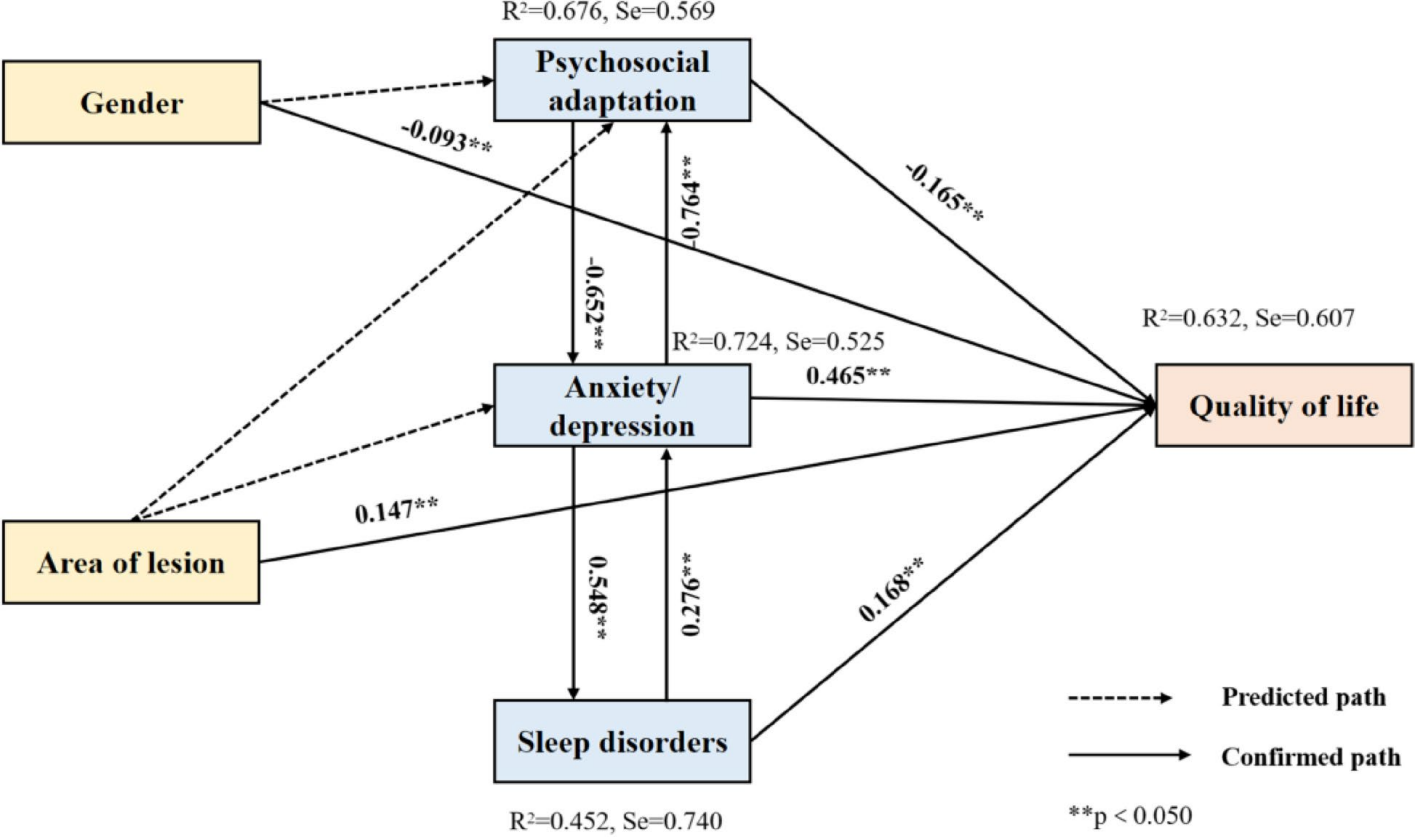
Path analysis of factors influencing QOL in people with psoriasis

According to previous theories and the literature, a path model was established for 5 variables in people with psoriasis, namely, anxiety/depression, lesion area, sleep disorders, psychosocial adaptation and sex. Four multivariate step-based analyses were carried out with anxiety/depression, sleep disorders, psychosocial adaptation and QOL as internal variables. Four internal variables could be effectively explained. The values of the explanatory variables were 0.621 (F(1056) = 61.020, *p* = 0.000) for QOL, 0.718 (F(2433) = 117.370, *p* = 0.000) for anxiety/depression, 0.440 (F(660) = 36.935, *p* = 0.000) for sleep disorders, and 0.669 (F(6886) = 93.556, *p* = 0.000) for psychosocial adaptation. The path analysis of the factors influencing QOL in people with psoriasis is shown in Fig. 1, which confirmed that 9 paths were consistent with the predicted path, and 3 paths were not confirmed.

## Discussion

A total of 96.76% of people with psoriasis self-reported impairment in QOL based on their DLQI scores. Analysis of the factors affecting QOL in people with psoriasis showed that anxiety/depression, lesion area, sleep disorders, psychosocial adaptation and sex were the main influencing factors. According to previous theories and the literature, a path model was established for 5 variables in people with psoriasis, showing their interaction and impact.

### Immutable factor affecting patients with psoriasis

Eight papers looked at the relationship between sex and QOL, and six of them showed that women had worse QOL than men [10, 11, 32]. Compared with men, women experience lower levels of happiness and greater levels of stress, loneliness, stigmatization, and sexual impairment [33]. The onset of psoriasis also often coincides with peak reproductive years for women, posing specific challenges for their treatment. Thus, while some studies have shown that men have significantly more severe psoriasis than women, they do not differ in their awareness of the severity of psoriasis symptoms, but they do differ in the degree of discomfort and QOL, which is consistent with the results of this study.

### Modifiable factors affecting people with psoriasis

Psoriasis patients with severe anxiety/depression have a lower QOL [34]. Higher anxiety/depression and lower QOL are common in patients with psoriasis, especially in women and patients with genital or joint lesions [14]. Some authors have found lower QOL and higher stress ratings in patients with extensive psoriasis, whereas Sampogna et al. reported a poor correlation between the extent of the disease and the effect on QOL [35–37]. Increased body surface area (BSA) add to the financial burden of treatment and indirectly lead to lower QOL [35]. The smaller the area of the lesion is, the better the QOL in people with psoriasis [38]. There were also associations between impaired QOL and skin lesion visibility, disease activity and severity. Skin discomfort and itchiness are thought to negatively affect QOL [10]. Psoriasis impacts various aspects of patients' QOL and is associated with sleep disturbances [23]. Chronic pruritus is often accompanied by sleep disturbances, which result in the disruption of social relationships, high rates of anxiety/depression, suicidal tendencies and lower QOL [18, 19]. People with psoriasis should be evaluated for sleep disturbances, itching, and anxiety/depression. And a specific assessment of sleep disorders using dedicated scores is necessary, especially since psoriasis can alter the physical and mental health of patients [22]. Reducing itching should be considered an important therapeutic goal, along with treatments aimed at reducing anxiety/depression [15]. The lower the level of anxiety/depression, the smaller the lesion area, the better the sleep or the higher the level of psychosocial adaptation in people with psoriasis, the higher their QOL is.

### Psychosocial interventions to improve the QOL of people with psoriasis

Psychosocial interventions can effectively affect various mechanisms in the pathogenesis of pruritus and improve the social function of patients with chronic skin pruritus [17]. Many psychosocial interventions have been shown to be effective, such as multidisciplinary teams [6, 27], educational interventions (therapeutic patient education, psychoeducational interventions, and self-management education) [6, 27], psychosocial interventions (cognitive and behavioural therapy (CBT), self-help, and peer-to-peer support programs) [27–29] and others [29] (training based on motivational interviews).

### Strengths and limitations

The study participants were recruited from the outpatients and inpatients of 3 hospitals, and the research results were representative. People with psoriasis recruited from a trained nurses. There was no conflict of interest between her and the subjects, which prevents some bias. Full anonymity is not possible because informed consent is required. And the use of self-reports might influence answers that show more "morally acceptable" intent. Due to the cross-sectional design, all study conclusions cannot prove causality between QOL and, for example, anxiety and depression.

## Conclusion

To improve QOL among people with psoriasis, attention should be given to anxiety/depression, lesion area, sleep disorders, psychosocial adaptation and sex differences. In addition to promoting the use of drugs, topical drugs and phototherapy to effectively control the progression of the disease, we should also pay attention to psychosocial interventions that can improve the QOL of people with psoriasis.

## Data Availability

All data generated or analysed during this study are included in this published article.
